# Developmental competence of IVF and SCNT goat embryos is improved by inhibition of canonical WNT signaling

**DOI:** 10.1371/journal.pone.0281331

**Published:** 2023-04-19

**Authors:** Marjan Sadeghi, Mohsen Rahimi Andani, Mehdi Hajian, Nafiseh Sanei, Reza Moradi-Hajidavaloo, Nasrin Mahvash, Farnoosh Jafarpour, Mohammad Hossein Nasr-Esfahani

**Affiliations:** 1 Department of Biology, Faculty of Science and Technology, ACECR Institute of Higher Education (Isfahan), Isfahan, Iran; 2 Department of Animal Biotechnology, Reproductive Biomedicine Research Center, Royan Institute for Biotechnology, ACECR, Isfahan, Iran; USP FZEA: Universidade de Sao Paulo Faculdade de Zootecnia e Engenharia de Alimentos, BRAZIL

## Abstract

The specific role of the canonical WNT/β-catenin signaling pathway during the preimplantation development of goat remains unclear. Our objective was to investigate the expression of β-CATENIN, one of the critical components of Wnt signaling pathway, in IVF embryos and compare it with SCNT embryos in goat. In addition, we evaluated the consequence of inhibition of β-catenin using IWR1. Initially, we observed cytoplasmic expression of β-CATENIN in 2 and 8–16 cell stage embryos and membranous expression of β-CATENIN in compact morula and blastocyst stages. Furthermore, while we observed exclusively membranous localization of β-catenin in IVF blastocysts, we observed both membranous and cytoplasmic localization in SCNT blastocysts. We observed that Inhibition of WNT signaling by IWR1 during compact morula to blastocyst transition (from day 4 till day 7 of *in vitro* culture) increased blastocyst formation rate in both IVF and SCNT embryos. In conclusion, it seems that WNT signaling system has functional role in the preimplantation goat embryos, and inhibition of this pathway during the period of compact morula to blastocyst transition (D4-D7) can improve preimplantation embryonic development.

## Introduction

Successful development of newly formed embryos [either IVF (*in vitro* fertilization) or SCNT embryos] is a complex process which requires precise and synchronized regulation of signaling pathways [1–6]. Any abnormal regulations of these mechanisms in embryos during early and late development results in an embryonic arrest or numerous phenotypically defects in the live offspring [4, 7, 8].

Even though the results of assisted reproductive techniques (ARTs) indicate that maternal signals are not essential during the early embryo development [9, 10], the importance of maternal signaling is revealed by higher defects in ART-derived embryos in terms of the transcriptome [10], proteome [11], metabolome [12] and epigenome [13] as compared to their *in vivo* counterparts. Furthermore, the higher proportion of defects in ART-derived embryos manifests itself through a lower pregnancy rate than that for *in vivo*-produced embryos [1, 14].

The highly orchestrated WNT signaling pathways were first identified for their critical role in the development of cancer [15, 16]. Then, later their function was recognized in a wide range of embryonic development processes, including body axis formation [17], maintenance of pluripotency [18, 19], the commitment of differentiation and the proliferation of cells [20, 21], migration of cells [22], and maintenance of hemostasis in adult tissues.

Wnt signaling pathway is a highly regulated and complex signaling pathway which is regulated by various WNT ligands, and these ligands interact and bind with a variety of receptors, including frizzled (FZD), receptor tyrosine kinase-like orphan receptor (ROR), receptor tyrosine kinase-related tyrosine kinase (RYK) and protein tyrosine kinase-7 (PTK7) and also co-receptors including low-density lipoprotein receptor-related proteins 5 and 6 (LRP5 and LRP6) [23–26].

Currently, three different WNT pathways are introduced to be activated upon WNT-receptor interaction [25, 27–30]. Canonical WNT/β-catenin signaling pathway is the well-identified downstream pathway and is strongly dependent on β-catenin [23].

The two other pathways are non-canonical pathways and are independent of β-catenin. A typical example of a non-canonical pathway is the planar cell polarity pathway (PCP). The other non-canonical pathway is the Ca^2+^ signaling pathway. Each pathway is activated by specific ligands and receptors that can be varied among different cell types or different physiological conditions, even in a defined cell type. These differences are related to multifaceted interactions between various ligands and receptors, and regulatory molecules in intercellular and intracellular microenvironments. Thus, specific ligand-receptor interactions can have various consequences in different cell types [26, 27, 29].

The canonical WNT/ß-Catenin pathway is initiated by the interacting WNT ligands with FRZ/LRP5 and LRP6 co-receptors. This complex recruits the dishevelled (Dvl), and subsequently Dvl traps the destruction complex containing Axin/APC/CK1/GSK-3β. This event prevents phosphorylation and degradation of β-catenin and then leads to the accumulation of β-catenin in the cytoplasm and translocation to the nucleus, where it activates transcription factors in the nucleus [31–33].

Canonical WNT/ß-Catenin is a maternally-derived pathway that seems to be essential for the preimplantation development of embryos [34]. Studies in the mouse indicate that this pathway is active as early as the two-cell stage around embryonic genome activation (EGA) [35]. In bovines, it has been shown that stimulation of WNT signaling using AMBMP (activator of WNT signaling pathway) during days 5 to 7 post-fertilization decreased the blastocyst formation rate. Supplementation of bovine embryo culture medium with DKK1 reversed the effect of AMPMP on blastocyst formation rate and CTNNB1 accumulation [36, 37]. In addition, some studies demonstrated that supplementation of bovine embryo culture medium with DKK1 from day 5 to 7 improved postimplantation developmental competence in terms of trophoblast elongation, secretion of interferon-τ in the uterus (day 15) and establishment of pregnancy after embryo transfer [38, 39].

While, up to our knowledge, there is no report about the role of WNT signaling pathway in the preimplantation development of goat, the results in bovine, led us to interpret the role of the WNT pathway by pharmacological inhibition of Wnt/β-catenin using a Tankyrase inhibitor, IWR1 in IVF- and SCNT derived embryos and investigating its effect on preimplantation embryo development.

## Material and methods

### Ethical considerations

All methods used in this study were performed under the Institutional Review Board and Institutional Ethical Committee of the Royan Institute guidelines and regulations (IR.ACECR.ROYAN.REC.1399.074). In addition, the slaughterhouse ovaries were transported to the embryology laboratory with the permission of the manager of the slaughterhouse and the agreement of the veterinary organization.

### Chemicals

All materials and media used in this study were obtained from Sigma-Aldrich Company (St Louis, MO, USA) and Gibco (Life Technologies, Rockville, MD, USA), respectively, unless otherwise indicated.

### Design of study and analysis endpoints

The experimental design of this study included the following steps:

In experiment 1, we first assessed the mRNA expression of *FRIZZLED* and *β-CATENIN* in 2 cell, 8–16 cell, compact morula and blastocyst embryos derived from IVF in goat. In this experiment, embryos were collected at each stage at a specific and defined time point. The 2 cell embryos were collected at 30-hour post insemination (hpi), 8–16 cell stage at 72 hpi, compact morula stage at 96 hpi and blastocyst stage at 144–156 hpi. The mRNA expression was assessed using real-time RT-PCR in three independent replications. In addition, the protein expression was assessed using β-CATENIN monoclonal antibody with immunocytochemistry (ICC) technique in stages mentioned above of goat IVF embryos in three independent replications.

Experiment 2 was designed to determine the effect of IWR1, a Wnt/β-catenin signaling inhibitor, on the development of goat IVF-treated embryos. First, the IVF embryos were treated with IWR1 (0, 1.25, 2.5 and 5 μM) for three days during *in vitro* culture (IVC) from day 5 (D5) to D7 (D5-D7). Then, we extended the treatment time from D4 to D7 (D4-D7). On day 7 post-fertilization the blastocyst rate, blastomere allocation (in D4-D7 group), protein expression of β-CATENIN using immunostaining (ICC) (in D4-D7 group) and mRNA expression of *OCT4*, *NANOG*, *CDX2*, and *β-CATENIN* using real-time RT-PCR (in D4-D7 group) were assessed.

Experiment 3 was carried out to compare the mRNA expression of *FRIZZLED* and *β-CATENIN* using real-time RT-PCR and protein expression of β-CATENIN using ICC between IVF and somatic cell nuclear transfer (SCNT) derived blastocysts.

Finally, in experiment 4, to assess the effect of supplementation of IVC with IWR1 on blastocyst rate of SCNT embryos, the SCNT embryos were treated with IWR1 (0, 1.25 and 5 μM) from D4 to D7 (D4-D7). Then, at day seven, blastocyst rate, blastomere allocation, protein expression of β-CATENIN and mRNA expression of *OCT4*, *NANOG*, *CDX2*, and *β-CATENIN* were assessed in the D4-D7 group.

### Preparation of IWR1 solution

Commercially available IWR1 (Santa Cruz Biotechnology, SC-295215) was dissolved in DMSO (10 mg in 489 μl DMSO), which was formulated in 50 mM stock solution and stored at -20°C. The desired working solutions including, 1.25, 2.5 and 5 μM, were made by diluting the stock solution with synthetic oviduct solution (SOF) [40]. In 5 μM IWR1 (the highest concentration of IWR1), the concentration of DMSO is 0.005%. We included the control-DMSO (0.005%) group to rule out the potential effect of DMSO on the blastocyst formation rate of treated embryos. While no detrimental effect was observed from DMSO (0.005%), we excluded this group from the data.

### Oocyte retrieval and in vitro maturation (IVM) of goat

In this study, goat ovaries were collected from two local slaughterhouses (Khomeini Shahr and Fasaran) and transferred to the embryology laboratory. After the arrival of the ovaries, the surrounding tissues were separated, and the ovaries were kept in the normal saline supplemented with penicillin/streptomycin at 15° C for overnight [41].

In the next day, after washing the ovaries, cumulus-oocyte complexes (COCs) were retrieved from follicles with diameter of 2–6 mm with a disposable 21-gauge needle connected to a vacuum pump (60–70 mm Hg). The aspiration medium was the HEPES-buffered tissue culture medium 199 (TCM199; HTCM199) supplemented with 10% fetal bovine serum (FBS) and heparin (10 μl/ml). The aspirated medium was transferred to a 10-cm culture dish, and only grade I and II oocytes were selected for *in vitro* maturation (IVM) [42].The process of IVM was according to our previous studies. In brief, after washing the grade I and II immature COCs in HTCM199 + 10% FBS, they were randomly cultured in a defined maturation medium in groups of 10 in 50 μl droplets. The composition of maturation medium was as we mentioned in our previously studies [41, 43]. The COCs were incubated for 20 h at 38.5°C in a humidified atmosphere containing 6.5% CO2.

### Sperm preparation and production of IVF embryos in goat

The IVF procedure was performed similar to our previous studies [44]. To this aim, frozen-thawed Sannen goat semen was used for fertilization. For isolation of motile sperms from immotile sperms, the swim down method was used. After isolating motile sperms, 10 matured COCs were co-incubated with 1 × 10^6^/ml motile sperms in droplets of fertilization medium at 38.5°C, 6.5% CO2 and humidified atmosphere. Eighteen hours post insemination, presumptive zygotes were mechanically denuded of cumulus cells, and cultured in synthetic oviduct fluid (SOF) supplemented with ITS (insulin, transferrin and selenium) and Myo-inositol (modified SOF: mSOF) without glucose and serum (mSOF-) for 3 days at 38.5°C, 6.5% CO2 and 5% O2 in humidified air under mineral oil. On Day 3, the cleavage rate was assessed. After that, the embryos were transferred to mSOF medium supplemented with charcoal stripped serum (mSOF^+^), and the blastocyst rate was evaluated on Day 7. The derived embryos at 2 cell, 8–16 cell, compact morula and blastocyst stages were used for evaluating the mRNA and protein expression of target gene(s) (experiments 1 and 3). In addition, in the periods mentioned above (Please see the experimental design section, experiment 2), the IVC medium (D5-D7 or D4-D7) was supplemented with IWR1 (0, 1.25, 2.5 and 5 μM) to assess the inhibition of WNT pathway on preimplantation development of IVF embryos.

### Preparation of goat ear fibroblast cells as somatic donor cells

Goat ear fibroblast cells (GEFs) were obtained from the Sannen breed, similar to our previous papers [45].The specimen from the skin of the ear was taken from a 1-month-old female Sannen goat. The presumptive G0 population of fibroblast donor cells were established by serum starvation (0.5% FBS) for 3–5 days. Fibroblasts at passages 3–4 were used for SCNT. Before use, donor cells were trypsinized and resuspended in HTCM199 + 0.5% FBS.

### SCNT procedure in goat

Twenty hours after IVM, cumulus cells were removed from matured COCs with hyaluronidase (300 IU/ml). Then, the zona pellucida was dissolved by treating with pronase (2.5 mg/ml) for up to 30 secs. A finely handmade pulled Pasteur pipette was used for enucleation of the matured oocytes [46]. Demecolcine treatment (4 μg/ml for 20 min) was used for facilitating manual enucleation and reducing injury during oocyte enucleation. Then, oocyte enucleation was carried out by positioning the oocytes so that the cytoplasmic extrusion that contains the MII chromosome mass is removed with the minimum amount of cytoplasm using a handmade oocyte enucleation pipette. Then, each fibroblast donor cell was attached to each enucleated oocyte using phytohemagglutinin (PHA). Then, two direct electric pulses with a time delay of 1 second (Cryologic^®^, Australia) were used for oocyte-cell fusion. Then, the oocyte-cell couplets were activated using ionomycin (5 μM) for 1 min and then cultured in 2 mM 6-DMAP at 38.5°C, 6.5% CO2, and maximum humidified atmosphere for 2 h under mineral oil. Finally, SCNT embryos were cultured in mSOF^+^ for 7 days at 38.5°C, 6.5% CO2, 5.5% O2, and humidified atmosphere under mineral oil [47]. The SCNT blastocysts were used for evaluating the mRNA and protein expression of *ß-CATENIN*/ß-CATENIN (experiment 3). In addition, the IVC medium of SCNT embryos was supplemented with IWR1 (0, 1.25 and 5 μM) during D4 to D7 (D4-D7) to assess the inhibition of WNT pathway on preimplantation development of SCNT embryos.

### Differential staining

The quality of the IVF and SCNT-derived blastocysts from various treatments was evaluated by differential staining allowing us to distinguish and quantify the blastomere allocation to trophectoderm (TE) and inner cell mass (ICM) that appeared pink or blue under UV light, respectively. Briefly, blastocysts at day 7 were washed in HTCM199 and 5 mg/mL bovine serum albumin (BSA). After that the blastocysts were treated with 0.5% Triton X-100 for 20 secs and then with 30 mg/mL propidium iodide for 30 secs. In the end, blastocysts were stained and fixed for 15 minutes in ice-chilled ethanol supplemented with 10 μg/ml Hoechst 33342. After mounting on the slide, the blastocysts were examined using a fluorescent microscope (Olympus BX51, Tokyo, Japan). At least 20 blastocysts (in total) in three replicates were used for differential staining.

### Immunocytochemistry staining for detection of ß-CATENIN

Embryos were washed three times in phosphate-buffered saline/polyvinyl alcohol (PBS/PVA), fixed in 4% Paraformaldehyde (PFA) for 20 min. After washing extensively, embryos were permeabilized with 1% Triton-X-100 for 30 min at RT. Then, the unspecific binding sites in embryos were blocked by treating with blocking solution (PBS/PVA containing 0.1% Triton-X-100 + 1% BSA + 10% goat serum). Embryos were then incubated for overnight at 4°C with ß-CATENIN primary antibody (Anti-B-catenin antibody (E5); sc-7963, Santa Cruz). An appropriate negative control was carried out by omitting the primary antibody and using mouse IgG (S1 Fig). After washing extensively, embryos were incubated with FITC conjugated goat anti-mouse IgG (Millipore; AP124F) for 1 hour at 37ºC in the dark. The nucleus was labelled by 1 μg/ ml Hoechst 33342 for 15 min at RT. Embryos were finally rinsed in PBS/PVA and placed on a slide containing mounting solution, covered with a coverslip, and observed with a fluorescence microscope (Olympus BX51, Tokyo, Japan). The images were captured through a sensitive camera (Olympus DP71). At least 20 blastocysts (in total) in three replicates were used for differential staining. The fluorescence intensity was assessed by Image J software (National Institutes of Health, Bethesda, MD) and then was normalized by the area in each individual embryo.

### Evaluation of mRNA expression

The mRNA expression of target genes (see Table 1) was analyzed in embryos. The 2 cell, 8-16-cell, compact morula and blastocyst embryos were collected 30-, 72-, 96- and 144-156-hour post insemination (hpi), respectively. All embryos were washed three times in PBS^-^, collected in pools of 20, frozen and stored at -80°C until RNA extraction. All embryo pools used for RNA extractions were collected and analyzed in triplicates. RNA extractions of the embryo pools were then performed using the RNeasy Plus Micro Kit (Qiagen, 74034) according to the manufacturer’s protocol. Total RNA was reverse transcribed using a cDNA Synthesis kit (Biotech rabbit) according to the manufacturer’s protocol. The quality and integrity of cDNA were checked using PCR and the housekeeping primer (*B-ACTIN*) as a reference gene. Real time PCR (qPCR) was performed using the SYBR Green Technology. Analysis of qPCR was performed in a 10 μl reaction volume by adding 1 μl cDNA in the PCR mix containing gene specific primers (250 nM final concentration, 0.5 μl for each forward and reverse primer) and 5 μl SYBR Green. qPCR conditions were 5 min at 95°C and 40 cycles of 20 seconds at 95°C and 20 seconds at specific annealing temperature for each specific paired primer. At the end of every reaction, a melt curve analysis was performed to ensure the specificity of the products. The mean cycle threshold (Ct) was calculated after performing three technical replicates for each sample. Relative expression was computed using mean Ct values which were normalized against *B-ACTIN*. Fold change in gene expression was calculated using 2-ΔΔCT. All the primers were designed by the Primer 3 program (http://primer3.ut.ee/), and their characteristics are listed in Table 1.

**Table 1:** List of primers used in this study for real-time PCR.

Symbol Gene	Forward Primer	Reverse Primer	Annealing Temp. (°C)	Length
*β-CATENIN*	AGTGGGCGGCATAGAGG	CACAGGTAGTCCGTAG	54	160
*CDX2*	CCCCAAGTGAAAACCAG	TGAGAGCCCCAGTGTG	56	144
*FRIZZLED*	ATTGCCTGCTACTTTTAC	TTAGTCTGGTTGTTCATT	54	89
*NANOG*	GATTCTTCCACAAGCCCT	TCATTGAGCACACACAGC	53	137
*OCT4*	GGAAAGGTGTTCAGCCA	ATTCTCGTTGTTGTCAGC	57	123
*β-ACTIN*	CCATCGGCAATGAGCGGT	CGTGTTGGCGTAGAGGTC	57	146

### Statistical analysis

All assessments were carried out at least three times. Data are presented as mean ± S.E.M. Statistical significance was set at *P*< 0.05. One-way analyses of variance (ANOVA) was applied to compare the effect of the treatments between various groups (α = 0.05), followed by the Tukey *post*-hoc test. The comparison of mRNA and protein expression of β-CATENIN between IVF and SCNT groups and also the mRNA expression of target genes in IVF and SCNT blastocysts derived from IWR1 (5 μM) treated groups compared to their control groups were analyzed by independent samples t-test. GraphPad Prism (v.6.0.1) and IBM SPSS program (v.23, NY, USA) were used for creating graphs and statistical analysis, respectively.

## Results

### Experiment 1: mRNA expression of FRIZZLED and β-CATENIN and protein expression of β-CATENIN in IVF preimplantation embryos in goat

**Fig 1:**
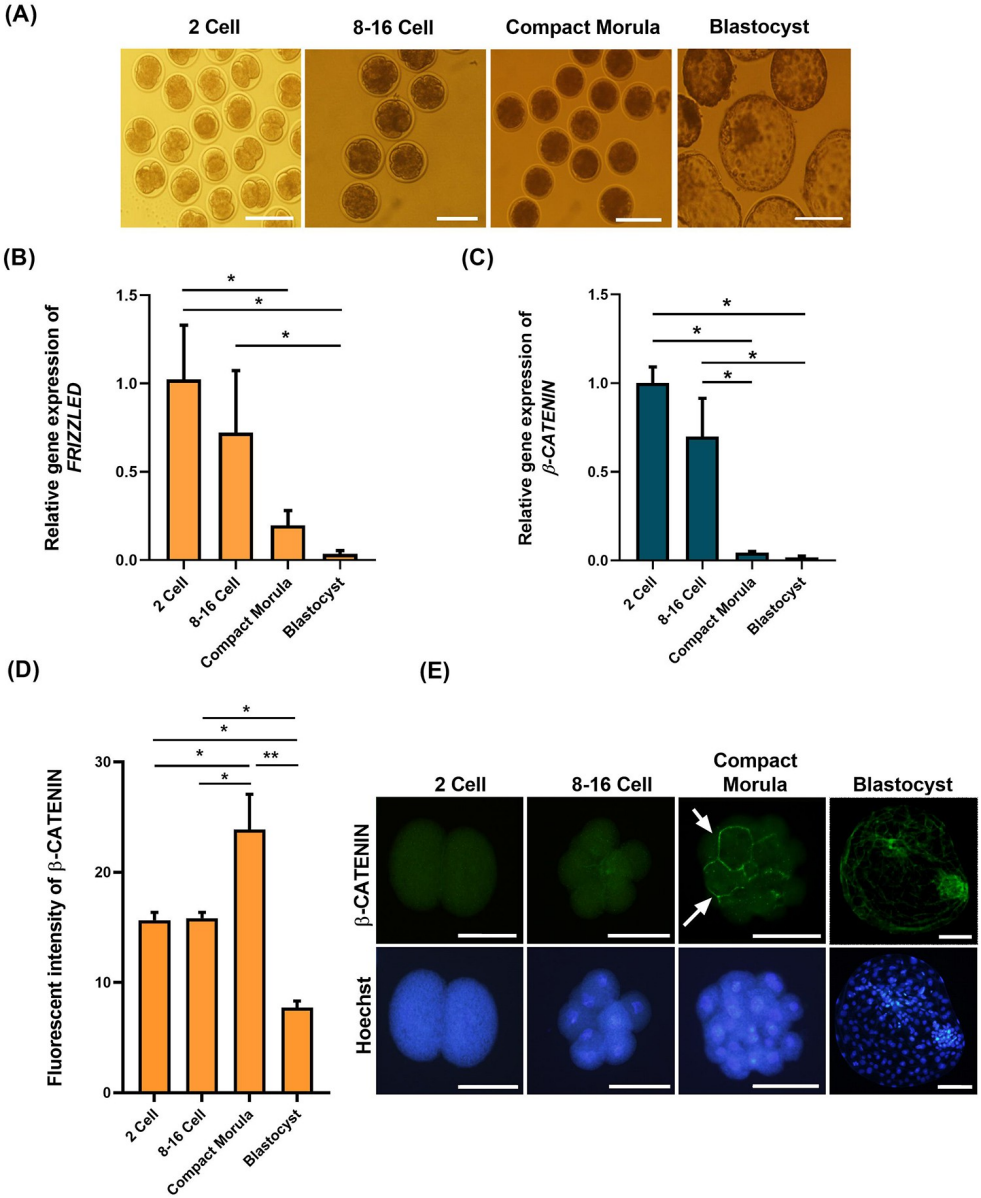
(A) The representative bright-field images of preimplantation development of IVF embryos including (from left to right) 2-cell, 8–16 cell, compact morula and blastocyst stages in goat. A relative pattern of mRNA expression for (B) *FRIZZLED* and (C) *β-CATENIN* during preimplantation embryo development of IVF embryos at the indicated developmental stages. (D) Quantification of the fluorescent intensity of β-CATENIN level in IVF embryos at the indicated developmental stages. (E) Representative immunofluorescence images of β-CATENIN in IVF embryos at the indicated developmental stages. Mean ± S.E.M. (standard error of the mean) values are from at least three independent experiments (* *P*< 0.05 and ** *P*< 0.01). Scale bars represent 100 μm.

In the first step of this experiment, we assessed the relative mRNA expression of *FRIZZLED* and *β-CATENIN*, two crucial components of the WNT signaling pathway, in IVF preimplantation goat embryos (2 cell. 8–16 cell, compact morula and blastocyst, Fig 1A) to demonstrate the status of this pathway using real-time RT-PCR. As depicted in Fig 1B and 1C, the relative mRNA expression of *FRIZZLED* and *β-CATENIN* had a decreasing pattern from 2 cell stage toward the blastocyst stage (Fig 1B and 1C, *P*< 0.05). The mRNA expression level of both genes was similar between 2 cell and 8–16 cell stage (*P*> 0.05) and also between the compact morula and blastocyst stages (*P*> 0.05), but it was significantly lower in compact morula and blastocyst stage as compared to 2 cell and 8–16 cell stage embryos (*P*< 0.05).

In the next step, we immunocytochemically assessed the protein expression of β-CATENIN, the main component of the WNT signaling pathway in preimplantation IVF goat embryos. The immunolabelling of β-CATENIN revealed cytoplasmic localization of β-CATENIN in 2-cell, 8–16 cell and compact morula stage (Fig 1E). Interestingly, we observed a shift from cytoplasmic to membrane localization in some blastomeres at the compact morula stage (Fig 1E, white arrows). Finally, we observed membrane localization of β-CATENIN at the blastocyst stage in all blastomeres (ICM and TE). Quantification of fluorescence intensity of FITC-labeled β-CATENIN revealed higher intensity in the compact morula stage as compared to 2-cell, 8–16 cell and blastocyst stage (*P*< 0.05, Fig 1D and 1E). In addition, the lower fluorescence intensity in the blastocyst stage was observed as compared to 2-cell, 8–16 cell and compact morula stage (*P*< 0.05, Fig 1D and 1E), which was in consistent with mRNA expression of *β-CATENIN* in blastocyst embryos.

### Experiment 2: The effect of IWR1 on developmental competence, blastomere allocation and expression of target genes in IVF goat embryos

**Fig 2:**
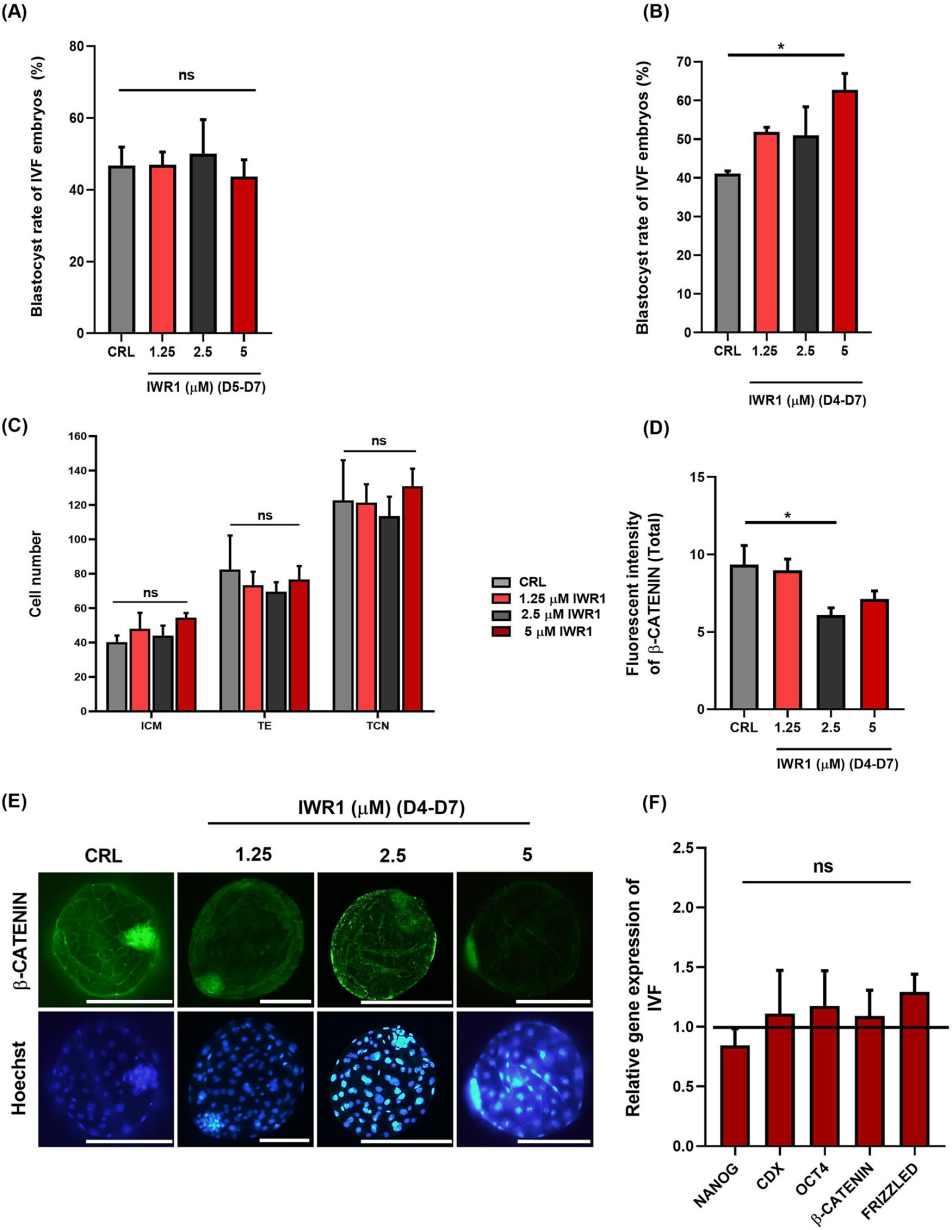
The effect of 1.25, 2.5 and 5 μM IWR1 during *in vitro* culture (IVC) from (A) D5 to D7 and (B) D4 to D7 in goat IVF embryos on blastocyst rate (/cleaved embryos). (C) The effect of IWR1 treatment on blastomere allocation of IVF treated embryos. (D) The effect of IWR1 treatment during D4-D7 on fluorescent intensity of β-CATENIN in IVF embryos. (E) Representative immunofluorescence images of β-CATENIN in IVF embryos following treatment with IWR1 during D4-D7. (F) The effect of 5 μM IWR1 during D4-D7 in goat IVF embryos on the relative expression of *NANOG*, *CDX2*, *OCT4* and *β-CATENIN*. Mean ± S.E.M. values are from at least three independent experiments (* *P*< 0.05 and ns *P*> 0.05). Scale bars represent 200 μm.

This experiment (Experiment 2) was carried out to assess if inhibition of canonical WNT signaling using IWR1 promoted the blastocyst formation of IVF goat embryos. Based on previous studies, we added various concentrations of IWR1 (1.25, 2.5 and 5 μM) [40] to the IVC medium on day 5 post-insemination (5 dpi) till 7 dpi (D5-D7 group) to assess the blastocyst developmental rate of IWR1 treated embryos. As depicted in Fig 2A (and also S1 Table), the addition of IWR1 at concentrations of 1.25, 2.5, and 5 μM did not change (*P*> 0.05) the blastocyst rate as compared to embryos cultured without IWR1.

In this study, treatment of goat IVF embryos was started at 5 dpi based on a study in bovine. While the compaction of goat embryos starts one day earlier (at 4 dpi) as compared to bovine embryos, we extended the time of treatment, and IWR1 was added to the IVC medium at 4 dpi) till 7 dpi (D4-D7 group) to assess the developmental competence of IWR1 treated embryos. As shown in Fig 2B (and also S2 Table), interestingly, we observed that the blastocyst rate was increased at 5 μM IWR1 (*P*< 0.05). While the blastocyst rate in the 1.25 and 2.5 μM groups was higher than the control group but it did not reach a significant level (Fig 2B, S3 Table *P*> 0.05).

Then, we assessed the effect of IWR1 on the quality of derived blastocyst. While the proportion of cleaved embryos that reach to blastocyst rate increased following treatment with IWR1, IWR1 did not significantly affect the ICM, TE and TCN (Fig 2D, *P*> 0.05).

In addition, we assessed the protein expression of β-CATENIN in derived blastocysts. As depicted in Fig 2D and 2E, we observed that 2.5 and 5 μM IWR1 significantly decreased the fluorescent intensity of β-CATENIN in the whole embryo at the blastocyst stage.

Then, blastocyst embryos at day 7 were assessed mRNA expression of target genes using real-time RT-PCR. The results (Fig 2F) revealed that mRNA expression of *OCT4*, *NANOG*, *CDX2*, and *β-CATENIN* remained unchanged after treatment with 5 μM IWR1 in D4-D7 group as compared to control group (*P*> 0.05).

### Experiment 3: Comparison of mRNA expression of FRIZZLED and β-CATENIN and protein expression of β-CATENIN in IVF and SCNT derived blastocysts in goat

**Fig 3:**
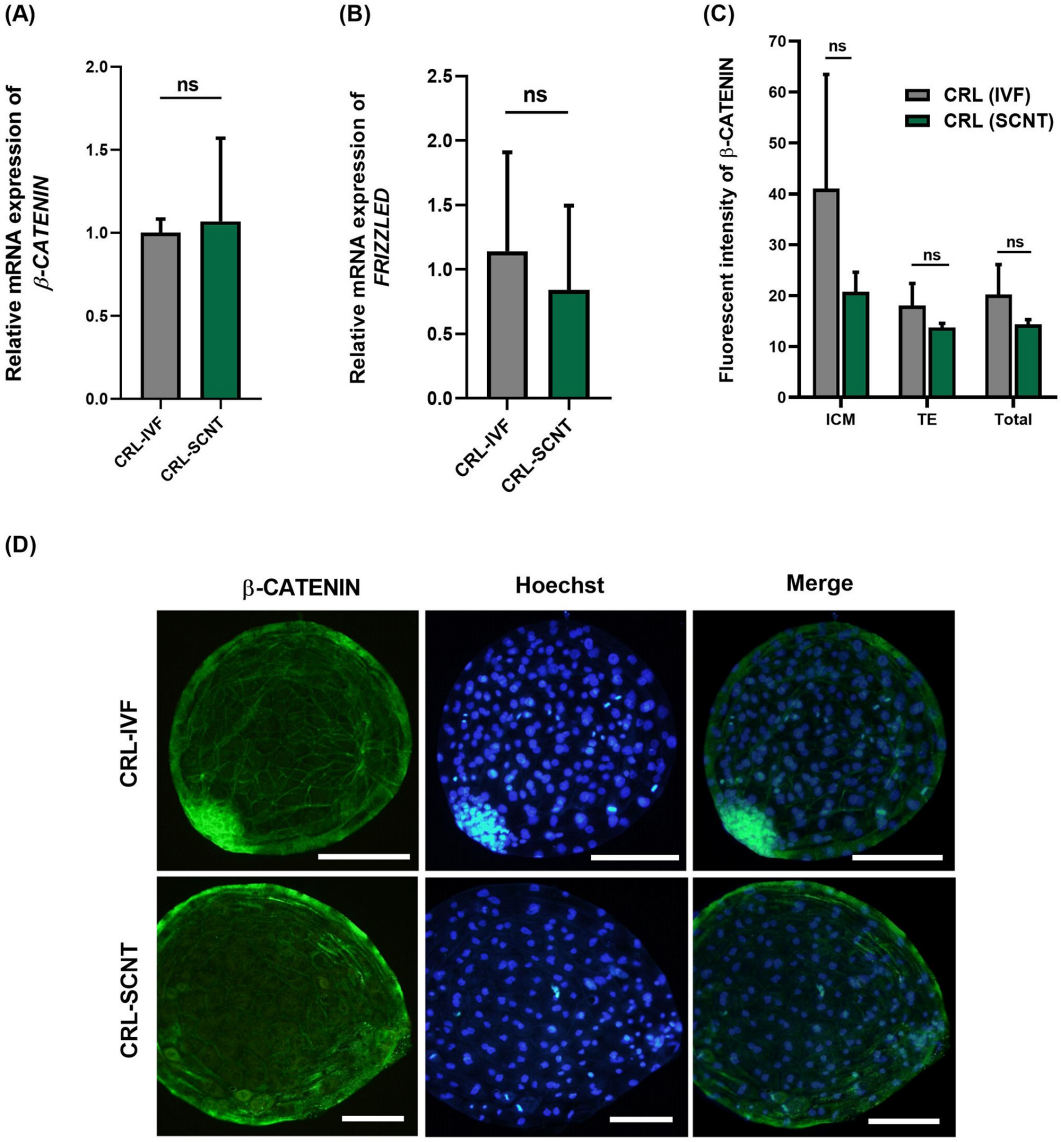
(A) Relative mRNA expression of (A) *β-CATENIN* and (B) *FRIZZLED* in IVF and SCNT blastocysts in goat. (C) Quantification of fluorescent intensity of β-CATENIN level in IVF and SCNT blastocysts. (D) Representative immunofluorescence images of β-CATENIN in IVF and SCNT blastocysts. Mean ± S.E.M. values are from at least three independent experiments (ns: *P*> 0.05). Scale bars represent 200 μm.

This experiment was designed to assess the relative mRNA expression of *FRIZZLED* and *β-CATENIN* in blastocysts derived from IVF and SCNT groups. As depicted in Fig 3A and 3B, the relative mRNA expression of *FRIZZLED* and *β-CATENIN* was similar between IVF and SCNT embryos at blastocyst stage (Fig 3A and 3B, *P*> 0.05).

In addition, the protein expression of β-CATENIN was assessed by immunocytochemistry in IVF and SCNT blastocysts. Quantification of fluorescence intensity of FITC-labeled β-CATENIN revealed a similar level of fluorescence intensity between IVF and SCNT embryos at the blastocyst stage (Fig 3C and 3D, *P*> 0.05). Interestingly, we observed that while the localization of β-CATENIN in IVF blastocysts is exclusively membranous, its localization in SCNT blastocysts is both membranous and cytoplasmic.

### Experiment 4: The effect of IWR1 on developmental competence, blastomere allocation and expression of target genes in SCNT goat embryos

**Fig 4:**
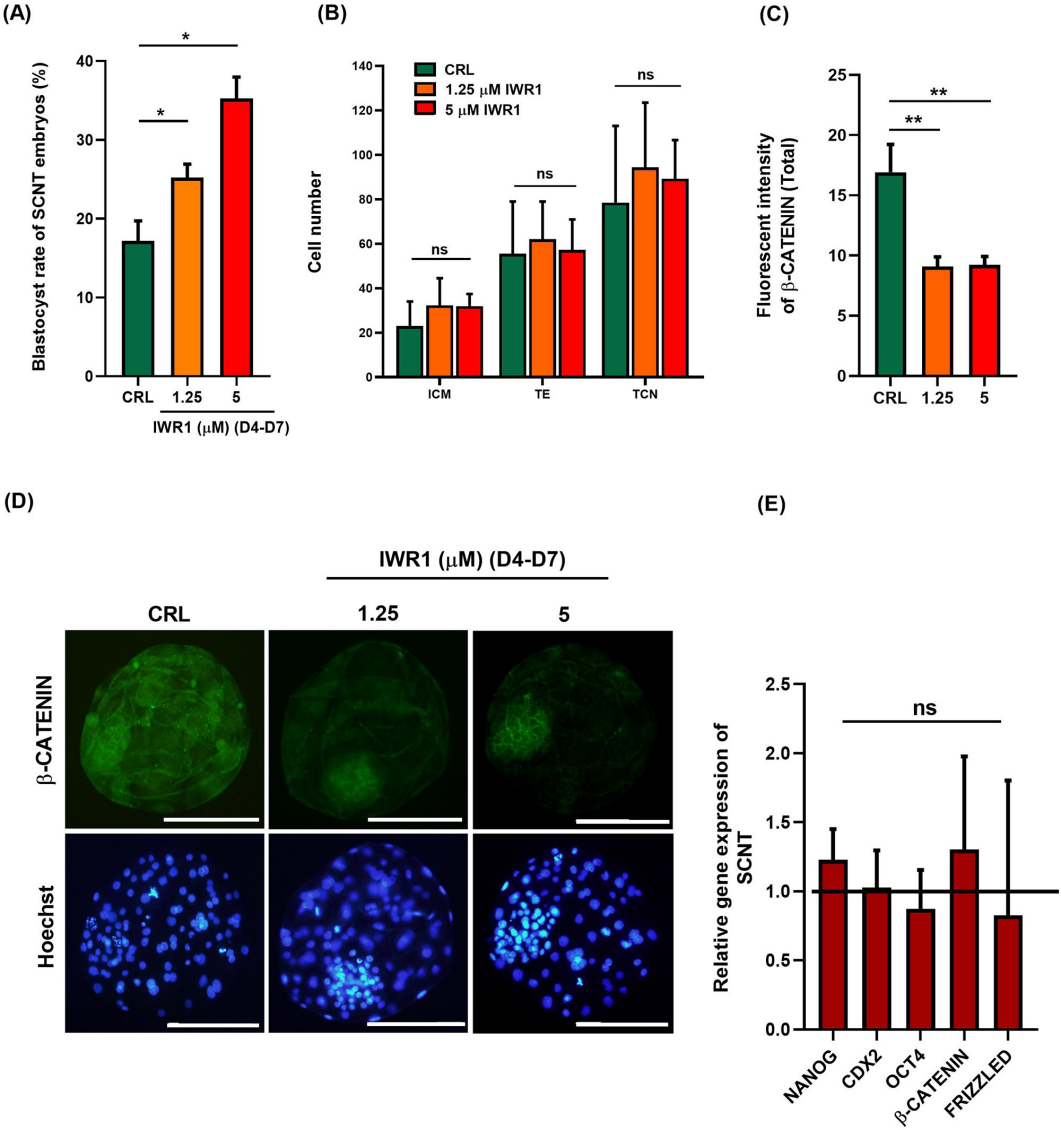
(A) The effect of 1.25 and 5 μM IWR1 during *in vitro* culture (IVC) from D4 to D7 in goat SCNT embryos on blastocyst rate (/cleaved embryos). (B) The effect of IWR1 treatment on blastomere allocation of SCNT treated embryos. (C) The effect of IWR1 treatment during D4-D7 on the fluorescent intensity of β-CATENIN in SCNT embryos. (D) Representative immunofluorescence images of β-CATENIN in SCNT embryos following treatment with IWR1 during D4-D7. (E) The effect of 5 μM IWR1 during D4-D7 in goat SCNT embryos on the relative expression of *NANOG*, *CDX2*, *OCT4* and *β-CATENIN*. Mean ± S.E.M. values are from at least three independent experiments (* *P*< 0.05 and ns *P*> 0.05). Scale bars represent 200 μm.

This experiment (Experiment 4) was carried out to assess if inhibition of canonical WNT signaling using IWR1 promoted the blastocyst formation of SCNT goat embryos. Based on results from IVF embryos (Experiment 2), we added 1.25 and 5 μM IWR1 to the IVC medium at day 4 post activation (4 dpa) till 7 dpa (SCNT D4-D7 group) to assess the blastocyst developmental rate. As depicted in Fig 4A (and also S3 Table), supplementation of culture medium with IWR1 caused a concentration-related increase (*P*< 0.05) in the percentage of cleaved embryos that developed to blastocyst stage at day 7.

Then, we assessed the effect of IWR1 on blastomere allocation at the blastocyst stage. While the proportion of cleaved embryos that reached to blastocyst stage increased following treatment with IWR1, IWR1 did not affect the ICM, TE and TCN (Fig 4B, *P*> 0.05).

Furthermore, we assessed if supplementation of IVC medium from D4 till D7 with IWR1 decreased the protein expression of β-CATENIN in derived blastocysts. With regard to this, we observed that both 1.25 and 5 μM IWR1 significantly decreased the fluorescent intensity of β-CATENIN in SCNT blastocysts (Fig 4C and 4D, *P*< 0.05). Interestingly, we observed that treatment of SCNT embryos with IWR1 shifted the localization pattern of β-CATENIN from both cytoplasmic and membranous to the only membranous pattern at the blastocyst stage (Fig 4C).

Then, assessing the mRNA expression of target genes in day 7 blastocysts revealed that, similar to IVF blastocysts (Fig 2F), mRNA expression of *OCT4*, *NANOG*, *CDX2*, and *β-CATENIN* in SCNT blastocysts remained unchanged after treatment with 5 μM IWR1 as compared to control group (Fig 4E, *P*> 0.05).

## Discussion

The canonical WNT pathway is one of the main signaling pathways involved in the early embryonic development of mammals, and little is known regarding its role in preimplantation embryos [48]. In general, the activity of the WNT/β-catenin pathway (localization of β-catenin in the nucleus) eventually leads to the expression of genes involved in proliferation, lineage commitment, and differentiation [49, 50]. On the other hand, membranous β-catenin (accumulation of β-catenin in the cell membrane) and its interaction with E-cadherin is involved in the cell-to-cell adhesion mechanism, which has a reciprocal relationship with nucleus β-catenin [51, 52]. Based on our results, it is likely that the WNT/β-catenin pathway is active around compaction stage, and becomes inactive at the blastocyst stage in IVF goat embryos. It is well-known that activation of the WNT/ß-catenin pathway and localization of ß-catenin in the nucleus increases the expression of pluripotency markers and decreases the expression of differentiation markers [50, 53]. So, it appears that increased protein expression of β*-*CATENIN is required for the process of compaction, and its subsequent reduction is required to maintain the state of pluripotency, at least in ungulates, to prevent ICM differentiation during dormancy or before implantation [54, 55]. Therefore, it is likely that the addition of IWR1 during days 4 to 7 may provide the opportunity for IVF goats embryos with lower developmental competency to undergo compaction and reduce the rate of premature or early differentiation. This proposition is consistent with the reduction in *β-*CATENIN in the whole embryo and in TE and ICM post IWR1 treatment. Previous studies have shown that different types of cell-to-cell adhesion, including Ca^2+^ dependent cell adhesion, gap junction, and tight junction, developed during the formation of compact morula and blastocyst embryos and is one of the perquisites for this stage [56–58]. With regard to this, it has been shown that tight junction is provided by E-cadherin/ß-catenin interaction in the cell membrane [59, 60], which is closely associated with the inactivation of the WNT/ß-catenin pathway in compact morula and blastocyst stages. Inactivation of the WNT/ß-catenin pathway during these stages leads to localization of ß-catenin in the cell membrane in association with E-cadherin and the establishment of tight junctions, which is necessary for the proper formation of compact morula and blastocyst stages. In addition, inactivation of the WNT/ß-catenin pathway leads to repression of pluripotency markers and mediates first lineage commitment in the compact morula and further blastocyst stages [50].

E-cadherin is associated with β-catenin, and these two proteins accumulate together in the cell membrane. This association stabilizes the β-catenin by inhibition of the binding of the destruction complex to the β-catenin [61]. It has been shown that the integrity of tight junction can be affected by degradation of intracellular part of cadherins [51]. A variety of pathways are required to work in a highly orchestrated manner to regulate a wide range of embryonic development processes. Simulation of embryonic development *in vitro* conditions, including IVF procedure, may dysregulate these pathways and hamper proper embryonic development. IWR1 is an inhibitor of the WNT pathway that acts intracellularly to inhibit Tankyrase (TNKS) and stabilize the AXIN, leading to an increase in the destruction of ß-catenin and the inactivation of this pathway [60]. We observed that treatment of IVF goat embryos from D5 to D7 did not improve blastocyst rate (Fig 2A, S1 Table. We used this time point for the treatment of embryos based in studies in bovines. We observed the inactivation of the WNT/ß-catenin pathway around the compact morula stage, and we started the treatment from day 5 at a time point when goat embryos passed the compaction stage and, it seems that the late inhibition of the WNT pathway in goat embryos did not improve blastocyst rate. So, we start the IWR1 treatment one day earlier than day 5. This change in the time of treatment improved the blastocyst rate in a dose-dependent manner and reached a significant level at 5 μM concentration. In addition, while we started the inhibition of the WNT/β-catenin pathway during the early stages of preimplantation development (day 1, after fertilization), the embryos did not benefit from this inhibition (S2 Fig), which demonstrates that an active WNT signaling pathway is necessary for the early development of goat embryos.

It has been shown that in *in vivo* condition, during compact morula formation, the endometrium express DKK1, which is an inhibitor for the WNT/β-catenin pathway [39, 62]. *In vitro* studies in bovine demonstrated that activating this pathway (using AMBMP) at day 5 decreased the blastocyst rate and reduced numbers of trophectoderm and inner cell mass cells. Further, they showed that DKK1 (inhibitor of the WNT/β-catenin pathway) could block the action of AMBMP and reverse its detrimental effect. However, DKK1, in the absence of AMBMP, did not improve the development, which is inconsistent with our result that may be related to species difference or the action of inhibitor of the WNT pathway (IWR1 vs DKK1) [37, 63]. These data confirm the necessity of WNT/β-catenin activation during the early stages of preimplantation development (before embryonic genome activation), which may mediate totipotency in developing embryos. In addition, these data reveal the need for inactivation of WNT/β-catenin in compact morula and blastocyst stages (after embryonic genome activation), which may establish the tight junctions between the blastomeres and start the first lineage commitment.

Additionally, we assessed the protein expression of the β-CATENIN in the treated embryos to monitor if treatment with IWR1 during D4-D7 of IVC, affects the expression of the β-CATENIN protein. The quantification of ICC images revealed effective inhibition of the WNT pathway by downregulation of the β-CATENIN protein in derived blastocysts. Next, we asked if inhibition of the WNT/β-catenin pathway affects blastomere allocation and, or gene expression. Our results revealed no difference in the blastomere allocation, pluripotency and trophectodermal markers in the derived blastocysts. Previous studies also showed that DKK1 (antagonist of the WNT/β-catenin pathway) did not affect the number of ICM or TE cells, suggesting that ICM proliferation is regulated through a mechanism independent of β-catenin, which needs to be elucidated [63]. In the next experiment, we assessed the mRNA expression of *FRIZZLED* and *β-CATENIN* and protein expression of *β-*CATENIN in goat IVF and SCNT blastocysts. The relative mRNA expression of *FRIZZLED* and *β-CATENIN* was similar between IVF and SCNT blastocysts. There are very limited studies about the status of this pathway in SCNT embryos. In contrast to our results, a recent study demonstrated the downregulation of DKK1 in SCNT compared to IVF blastocysts in buffalo which indicates the higher activity of the WNT/β-catenin pathway in SCNT blastocysts in buffalo as compared to their IVF counterparts [64]. Then, we immunocytochemically assessed the protein expression of β-CATENIN in IVF and SCNT blastocysts. While quantification of fluorescence intensity of FITC-labeled β-CATENIN revealed a similar level between IVF and SCNT blastocysts but the pattern of localization of β-CATENIN was different in IVF and SCNT embryos. IVF blastocysts revealed exclusively membranous localization, but SCNT blastocysts showed more cytoplasmic localization and less membranous localization, which maybe indicate higher activity of WNT/β-catenin in SCNT blastocysts in goat. Active WNT/β-catenin pathway in SCNT blastocysts may indicate weakened tight junctions and a delay in lineage commitment which both may hamper proper preimplantation and postimplantation development of SCNT embryos.

So, in the following experiment, similar to IVF embryos, SCNT embryos were treated with IWR1 from 4 dpa till 7 dpa. Similar to IVF embryos, we observed a higher blastocyst rate in IWR1 treated embryos. In addition, ICC images showed that IWR1 treatment changed the localization of β-CATENIN and exhibited only a membranous pattern, which is an indicator of the inactivation of the WNT/β-catenin pathway in SCNT blastocysts. Consequently, we hypothesized that maybe extended treatment of SCNT embryos with IWR1 during all days of IVC (D0-D7) may increasingly enhance the blastocyst rate. Interestingly, we observed that prolonged treatment of SCNT embryos (D0-D7) with IWR1 insignificantly decreased the blastocyst rate (S3 Fig) that, again, similar to the results of IVF embryos, indicates the necessity of WNT/β-catenin activation during early embryonic development (before EGA) which guarantees the totipotency and early embryonic development in growing embryos. Comparable to IVF embryos, inhibition of this pathway neither changed the blastomere allocation nor the expression of assessed genes at the mRNA level.

## Conclusion

Taken together, our data revealed activation of the WNT/β-Catenin pathway in the early stage of preimplantation development of IVF goat embryos and inactivation of this pathway around compact morula and blastocyst embryos. In addition, we demonstrated that supplement of IVC medium with IWR1, an inhibitor of the canonical WNT signaling pathway, before EGA but not after EGA promotes blastocyst formation rate in both IVF and SCNT goat embryos. Furthermore, IWR1 changed the localization of β-catenin from cytoplasmic to the membranous pattern in SCNT goat embryos. It is hypothesized that the action of IWR1 modulates WNT/β-catenin signaling in developing embryos in goat and may accelerate further development during postimplantation.

## Supporting information

S1 Fig Negative control for immunocytochemistry was carried out by omitting the primary antibody (ß-CATENIN) and using mouse IgG as primary antibody. Scale bars represent 200 μm.
(TIF)

S2 Fig The effect of 1.25, 2.5 and 5 μM IWR1 during *in vitro* culture (IVC) from D1 to D7 in goat IVF embryos on blastocyst rate (/cleaved embryos). (TIF)

S3 Fig The effect of 1.25 and 5 μM IWR1 during *in vitro* culture (IVC) from D0 to D7 in goat SCNT embryos on blastocyst rate (/cleaved embryos). (TIF)

S1 Table Evaluation of various concentrations of IWR1 on developmental competence of goat IVF embryos from D5 post insemination to D7 post insemination. At least three replications were performed for each treatment. Developmental rates of treated embryos were monitored as cleavage and blastocyst rates at day 3 and 7, respectively. Within a column, developmental rates with similar superscripts are not significantly different from each other (*P*> 0.05).
(DOCX)

S2 Table Evaluation of various concentrations of IWR1 on developmental competence of goat IVF embryos from D4 post insemination to D7 post insemination. At least three replications were performed for each treatment. Developmental rates of treated embryos were monitored as cleavage and blastocyst rates at day 3 and 7, respectively. Within a column, developmental rates with different superscripts (a and b) are significantly different from each other (P< 0.05).
(DOCX)

S3 Table Evaluation of various concentrations of IWR1 on developmental competence of goat SCNT embryos from D4 post activation to D7 post activation. At least three replications were performed for each treatment. Developmental rates of treated embryos were monitored as cleavage and blastocyst rates at day 3 and 7, respectively. Within a column, developmental rates with different superscripts (a, b and c) are significantly different from each other (P< 0.05).
(DOCX)
