# Supplementary figures and images for: Developmental competence of IVF and SCNT goat embryos is improved by inhibition of canonical WNT signaling
Source: PLoS One. 2023 Apr 19;18(4):e0281331. doi: 10.1371/journal.pone.0281331 (PMC10115261; doi:10.1371/journal.pone.0281331)

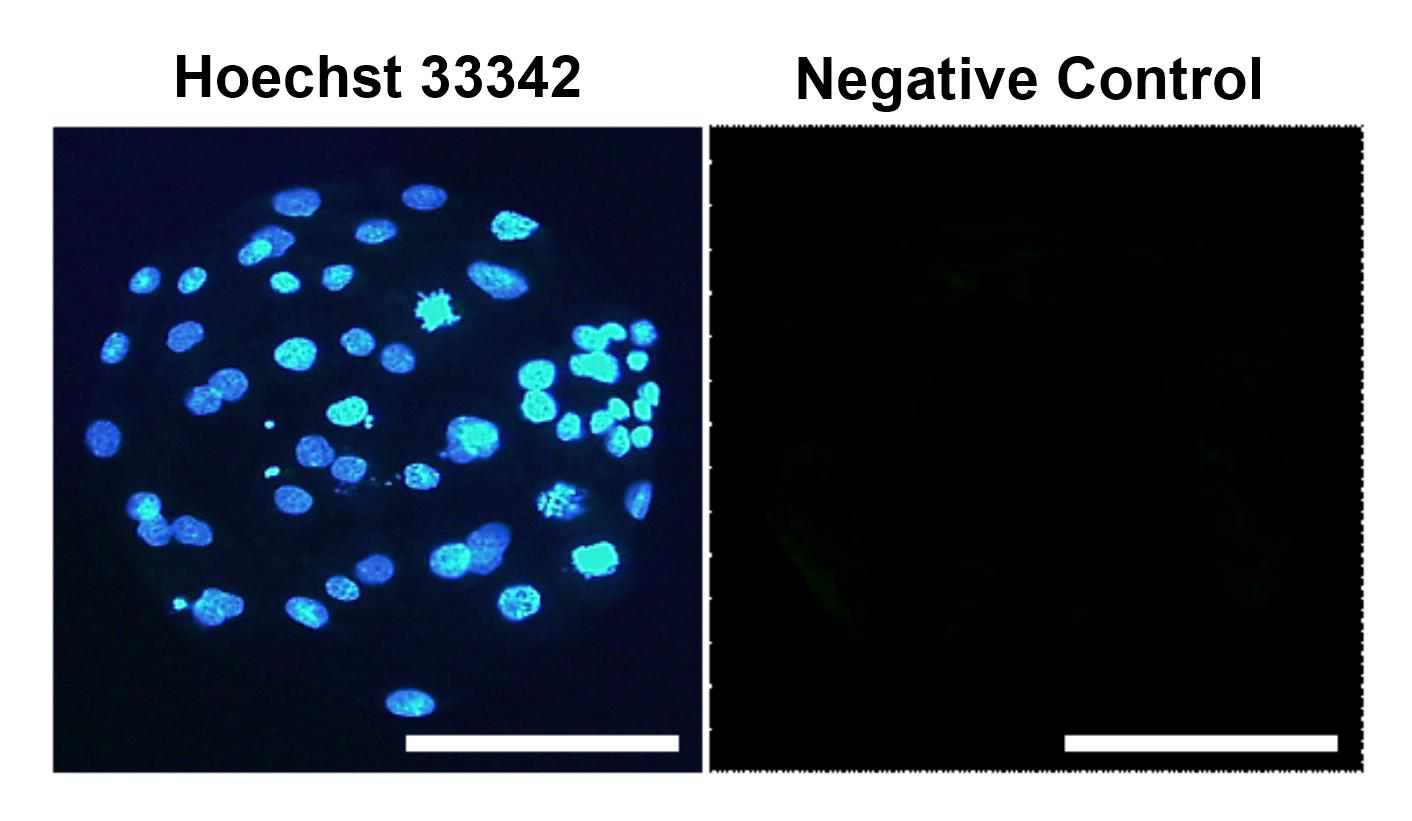

Supplement: S1 Fig — Scale bars represent 200 μm. (TIF) [file pone.0281331.s001.tif]

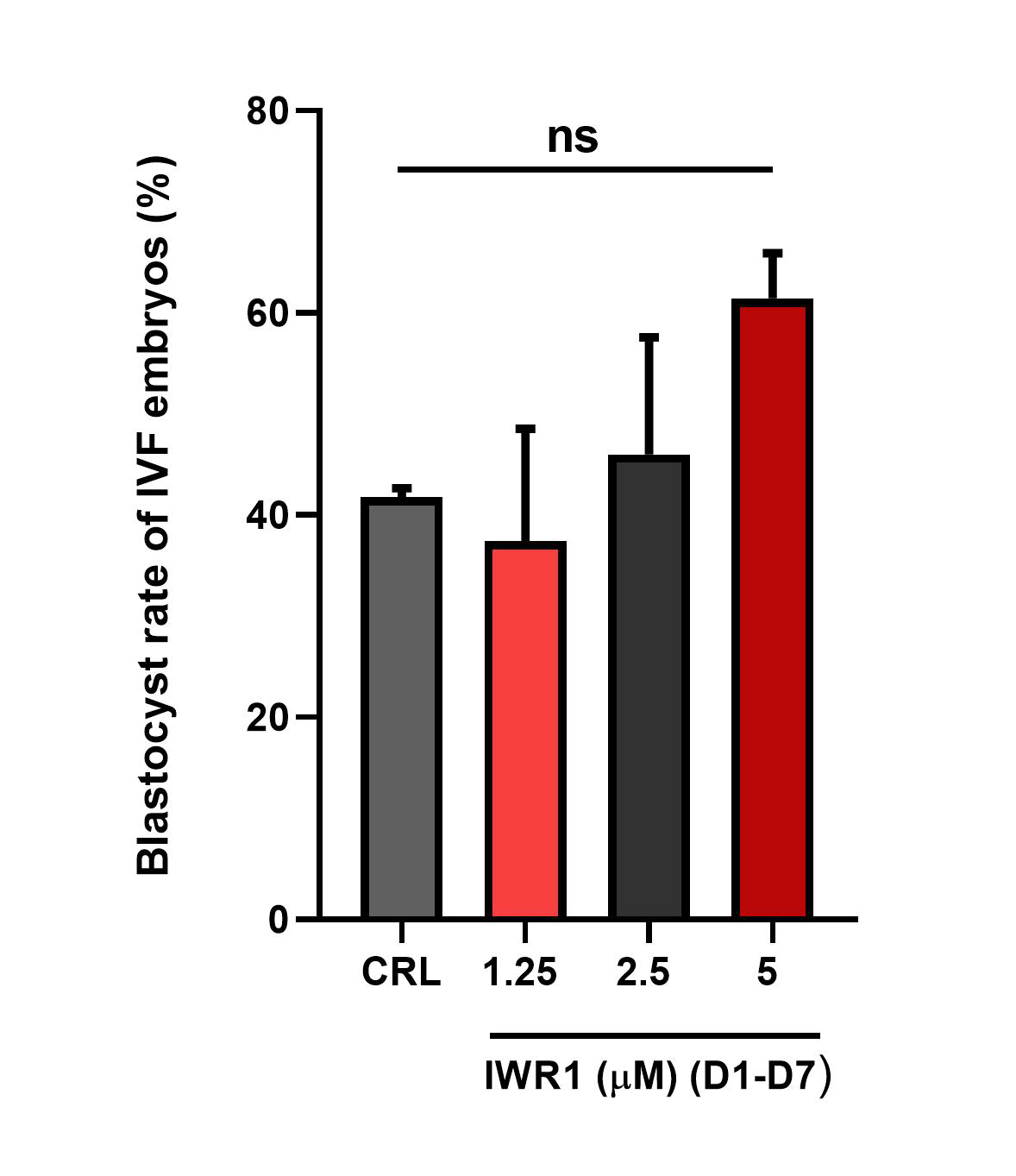

Supplement: S2 Fig — (TIF) [file pone.0281331.s002.tif]

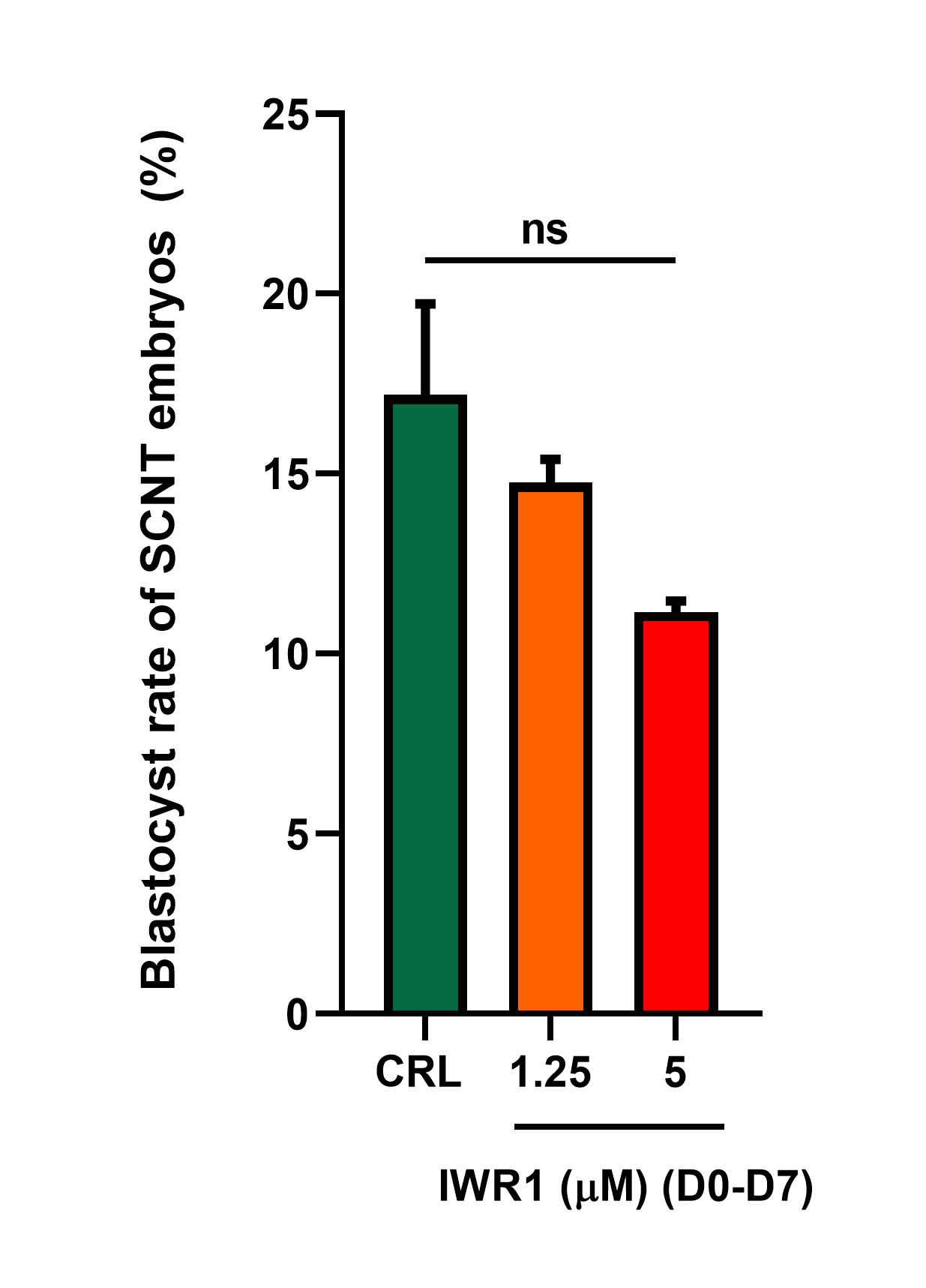

Supplement: S3 Fig — (TIF) [file pone.0281331.s003.tif]
