# Supplementary material for: Developmental competence of IVF and SCNT goat embryos is improved by inhibition of canonical WNT signaling
Source: PLoS One. 2023 Apr 19;18(4):e0281331. doi: 10.1371/journal.pone.0281331 (PMC10115261; doi:10.1371/journal.pone.0281331)
Supplement: S1 Table — At least three replications were performed for each treatment. Developmental rates of treated embryos were monitored as cleavage and blastocyst rates at day 3 and 7, respectively. Within a column, developmental rates with similar superscripts are not significantly different from each other (P> 0.05). (DOCX) [file pone.0281331.s004.docx]

| Table S1. Evaluation of various concentrations of IWR1 on developmental competence of goat IVF embryos from D5 post insemination to D7 post insemination | | | | |
| --- | --- | --- | --- | --- |
| Group | No. of COCs | No. of presumptive zygotes | No. of cleaved embryos (Mean ± S.E.M. %) | No. of blastocysts (Mean± S.E.M. %) |
| Control | 220 | 208 | 184 (88.46 ± 4.84) a | 86 (46.82 ± 5.08) a |
| 1.25 μM IWR1 | 230 | 214 | 162 (76.28 ± 6.31) a | 68 (47.35 ± 4.12) a |
| 2.5 μM IWR1 | 200 | 190 | 168 (88.42 ± 7.21) a | 84 (50.58 ± 10.32) a |
| 5 μM IWR1 | 210 | 196 | 175 (85.61 ± 4.28) a | 86 (43.67 ± 4.67) a |

At least three replications were performed for each treatment. Developmental rates of treated embryos were monitored as cleavage and blastocyst rates at day 3 and 7, respectively. Within a column, developmental rates with similar superscripts are not significantly different from each other (*P*> 0.05).
