# Supplementary material for: Developmental competence of IVF and SCNT goat embryos is improved by inhibition of canonical WNT signaling
Source: PLoS One. 2023 Apr 19;18(4):e0281331. doi: 10.1371/journal.pone.0281331 (PMC10115261; doi:10.1371/journal.pone.0281331)
Supplement: S2 Table — At least three replications were performed for each treatment. Developmental rates of treated embryos were monitored as cleavage and blastocyst rates at day 3 and 7, respectively. Within a column, developmental rates with different superscripts (a and b) are significantly different from each other (P< 0.05). (DOCX) [file pone.0281331.s005.docx]

| Table S2. Evaluation of various concentrations of IWR1 on developmental competence of goat IVF embryos from D4 post insemination to D7 post insemination. | | | | |
| --- | --- | --- | --- | --- |
| Group | No. of COCs | No. of presumptive zygotes | No. of cleaved embryos (Mean ± S.E.M. %) | No. of blastocysts (Mean± S.E.M. %) |
| Control | 620 | 590 | 421 (71.35 ± 3.46) a | 173 (41.12 ± 2.66) a |
| 1.25 μM IWR1 | 312 | 296 | 220 (74.32 ± 4.12) a | 114 (51.84 ± 3.23) ab |
| 2.5 μM IWR1 | 265 | 251 | 164 (65.33 ± 2.38) a | 84 (51.35 ± 7.34) ab |
| 5 μM IWR1 | 315 | 298 | 182 (61.07 ± 3.75) a | 114 (62.73 ± 4.23) b |

At least three replications were performed for each treatment. Developmental rates of treated embryos were monitored as cleavage and blastocyst rates at day 3 and 7, respectively. Within a column, developmental rates with different superscripts (a and b) are significantly different from each other (*P*< 0.05).
