# Supplementary material for: Developmental competence of IVF and SCNT goat embryos is improved by inhibition of canonical WNT signaling
Source: PLoS One. 2023 Apr 19;18(4):e0281331. doi: 10.1371/journal.pone.0281331 (PMC10115261; doi:10.1371/journal.pone.0281331)
Supplement: S3 Table — At least three replications were performed for each treatment. Developmental rates of treated embryos were monitored as cleavage and blastocyst rates at day 3 and 7, respectively. Within a column, developmental rates with different superscripts (a, b and c) are significantly different from each other (P< 0.05). (DOCX) [file pone.0281331.s006.docx]

| Table S3. Evaluation of various concentrations of IWR1 on developmental competence of goat SCNT embryos from D4 post activation to D7 post activation. | | | | |
| --- | --- | --- | --- | --- |
| Group | No. of COCs | No. of reconstructed oocytes | No. of cleaved embryos (Mean ± S.E.M. %) | No. of blastocysts (Mean± S.E.M. %) |
| Control | 580 | 351 | 309 (88.03 ± 4.37) a | 53 (17.19 ± 2.52) a |
| 1.25 μM IWR1 | 580 | 246 | 218 (88.61 ± 6.42) a | 55 (25.23 ± 1.69) b |
| 5 μM IWR1 | 360 | 220 | 182 (82.72 ± 5.37) a | 64 (35.25 ± 2.73) c |

At least three replications were performed for each treatment. Developmental rates of treated embryos were monitored as cleavage and blastocyst rates at day 3 and 7, respectively. Within a column, developmental rates with different superscripts (a, b and c) are significantly different from each other (*P*< 0.05).
